# 2-Amino-5-methyl­pyridinium 2-hy­droxy-5-chloro­benzoate

**DOI:** 10.1107/S160053681205101X

**Published:** 2012-12-22

**Authors:** Kaliyaperumal Thanigaimani, Abbas Farhadikoutenaei, Suhana Arshad, Ibrahim Abdul Razak

**Affiliations:** a School of Physics, Universiti Sains Malaysia, 11800 USM, Penang, Malaysia; b Department of Physics, Faculty of Science, University of Mazandaran, Babolsar, Iran

## Abstract

In the 5-chloro­salicylate anion of the title salt, C_6_H_9_N_2_^+^·C_7_H_4_ClO_3_^−^, an intra­molecular O—H⋯O hydrogen bond with an *S*(6) graph-set motif is observed and the dihedral angle between the benzene ring and the –CO_2_ group is 1.6 (6)°. In the crystal, the protonated N atom and the 2-amino group of the cation are hydrogen bonded to the carboxyl­ate O atoms *via* a pair of N—H⋯O hydrogen bonds, forming an *R*_2_^2^(8) ring motif. The crystal structure also features N—H⋯O and weak C—H⋯O inter­actions, resulting in a layer parallel to (10-1).

## Related literature

For details of non-covalent inter­actions, see: Desiraju (2007 ▶); Aakeroy & Seddon (1993 ▶). For background to the chemistry of substituted pyridines, see: Pozharski *et al.* (1997 ▶); Katritzky *et al.* (1996 ▶). For related structures, see: Nahringbauer & Kvick (1977 ▶); Raza *et al.* (2010 ▶); Thanigaimani *et al.* (2012*a* ▶,*b* ▶). For hydrogen-bond motifs, see: Bernstein *et al.* (1995 ▶). For bond-length data, see: Allen *et al.* (1987 ▶). For the stability of the temperature controller used for the data collection, see: Cosier & Glazer (1986 ▶).
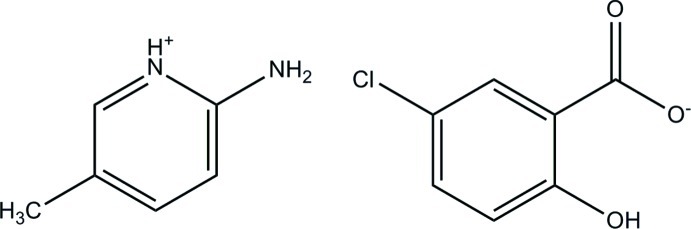


## Experimental

### 

#### Crystal data

C_6_H_9_N_2_^+^·C_7_H_4_ClO_3_^−^
*M* *_r_* = 280.70
Monoclinic, 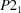

*a* = 9.004 (7) Å
*b* = 5.767 (5) Å
*c* = 12.617 (10) Å
β = 90.415 (16)°
*V* = 655.2 (9) Å^3^
*Z* = 2
Mo *K*α radiation
μ = 0.30 mm^−1^
*T* = 100 K
0.46 × 0.18 × 0.07 mm

#### Data collection

Bruker SMART APEXII Duo CCD area-detector diffractometer
Absorption correction: multi-scan (*SADABS*; Bruker, 2009 ▶) *T*_min_ = 0.877, *T*_max_ = 0.979
4785 measured reflections
2204 independent reflections
1539 reflections with *I* > 2σ(*I*)
*R*_int_ = 0.061

#### Refinement

*R*[*F* ^2^ > 2σ(*F* ^2^)] = 0.073
*wR*(*F* ^2^) = 0.201
*S* = 1.01
2204 reflections
189 parameters
2 restraints
H atoms treated by a mixture of independent and constrained refinement
Δρ_max_ = 0.47 e Å^−3^
Δρ_min_ = −0.44 e Å^−3^
Absolute structure: Flack (1983 ▶), 941 Friedel pairs
Flack parameter: 0.09 (16)

### 

Data collection: *APEX2* (Bruker, 2009 ▶); cell refinement: *SAINT* (Bruker, 2009 ▶); data reduction: *SAINT*; program(s) used to solve structure: *SHELXTL* (Sheldrick, 2008 ▶); program(s) used to refine structure: *SHELXTL*; molecular graphics: *SHELXTL*; software used to prepare material for publication: *SHELXTL* and *PLATON* (Spek, 2009 ▶).

## Supplementary Material

Crystal structure: contains datablock(s) global, I. DOI: 10.1107/S160053681205101X/is5232sup1.cif

Structure factors: contains datablock(s) I. DOI: 10.1107/S160053681205101X/is5232Isup2.hkl

Supplementary material file. DOI: 10.1107/S160053681205101X/is5232Isup3.cml

Additional supplementary materials:  crystallographic information; 3D view; checkCIF report

## Figures and Tables

**Table 1:** Hydrogen-bond geometry (Å, °)

*D*—H⋯*A*	*D*—H	H⋯*A*	*D*⋯*A*	*D*—H⋯*A*
O3—H1*O*3⋯O2	0.92 (6)	1.73 (7)	2.512 (6)	141 (6)
N1—H1*N*1⋯O2^i^	0.94 (6)	1.76 (6)	2.683 (7)	166 (5)
N2—H1*N*2⋯O1^i^	0.85 (5)	1.96 (5)	2.793 (8)	165 (4)
N2—H2*N*2⋯O1^ii^	0.85 (6)	2.04 (7)	2.811 (6)	152 (7)
C8—H8*A*⋯O3^iii^	0.95	2.58	3.425 (7)	148

Symmetry codes: (i) 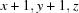; (ii) 
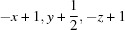
; (iii) 
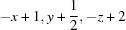
.

**Fig. 1.**
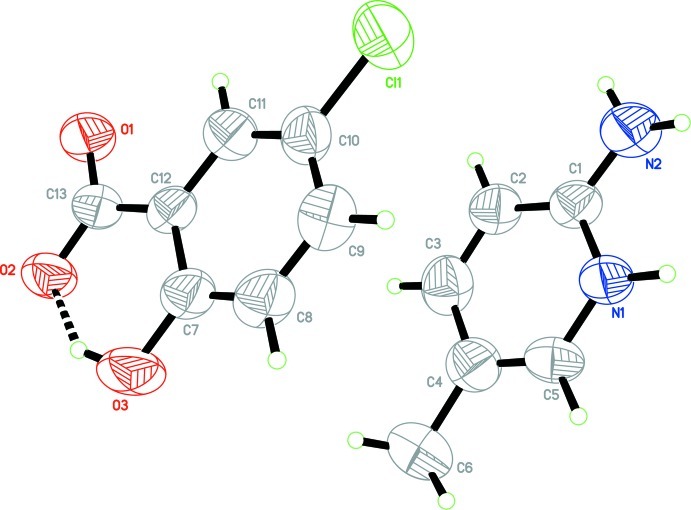
The molecular structure of the title compound with atom labels with 50% probability displacement ellipsoids.

**Fig. 2.**
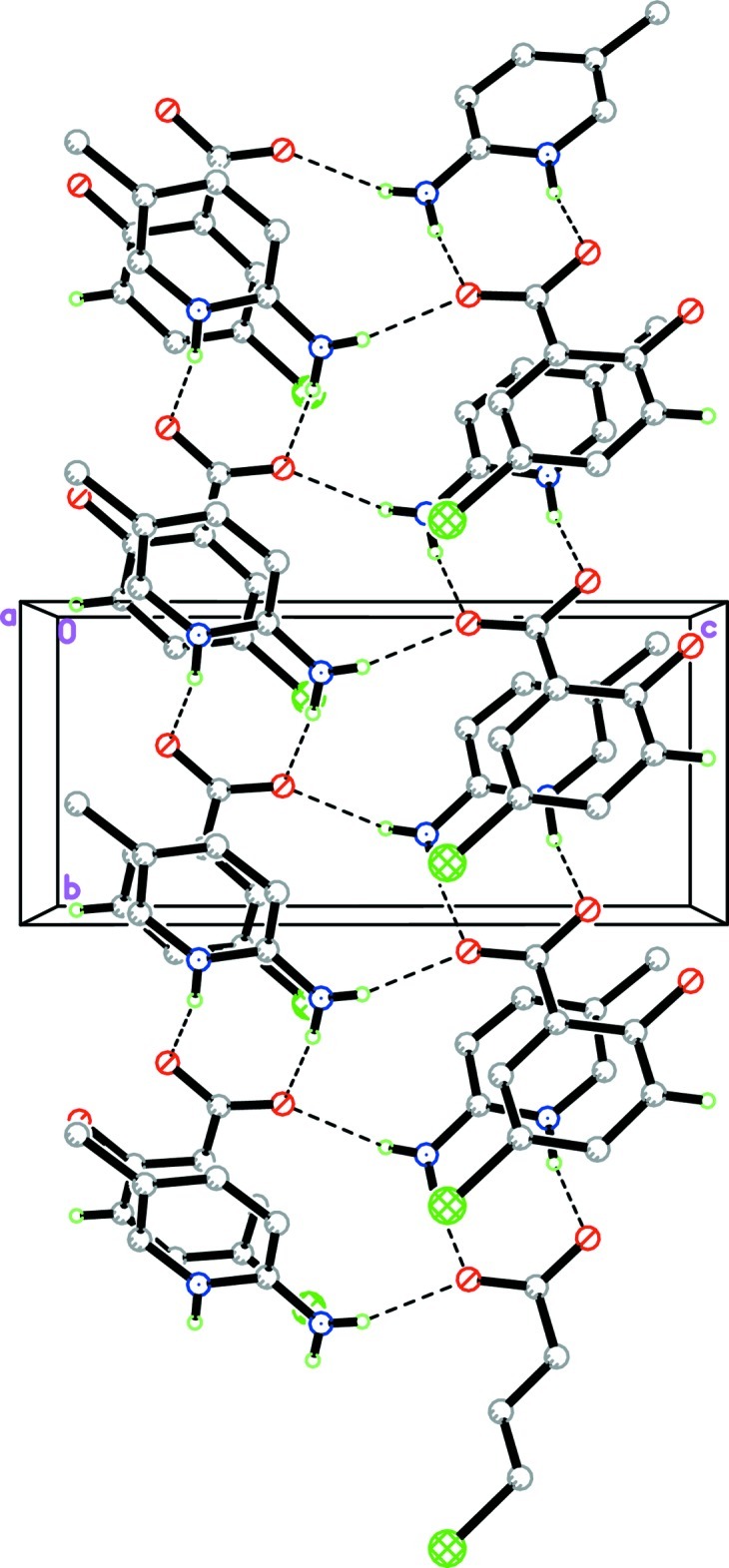
The crystal packing of the title compound. The H atoms not involved in the intermolecular interactions (dashed lines) have been omitted for clarity.

## supplementary crystallographic information

### Comment

Supramolecular architectures assembled *via* various delicate noncovalent interactions such as hydrogen bonds, π–π stacking and electrostatic interactions, *etc*., have attracted intense interest in recent years because of their fascinating structural diversity and potential applications for functional materials (Desiraju, 2007). Especially, the application of intermolecular hydrogen bonds is a well known and efficient tool in the field of organic crystal design owing to its strength and directional properties (Aakeroy & Seddon, 1993). Pyridine and its derivatives play an important role in heterocyclic chemistry (Pozharski *et al.*, 1997; Katritzky *et al.*, 1996). The are often involved in hydrogen-bond interactions. In order to study potential hydrogen bonding interactions, as part of our ongoing studies on pyridine derivatives (Thanigaimani *et al.*, 2012*a*,*b*), the crystal structure determination of the title compound (I) was carried out.

The asymmetric unit (Fig. 1) contains one 2-amino-5-methylpyridinium cation and one 5-chlorosalicylate anion. The proton transfers from the one of the carboxyl group oxygen atom (O2) to atom N1 of 2-amino-5-methylpyrimidine resulted in the widening of C1—N1—C5 angle of the pyridinium ring to 121.0 (5)°, compared to the corresponding angle of 117.4 (3)° in neutral 2-amino-5-methylpyridine (Nahringbauer & Kvick, 1977). The 2-amino-5-methylpyridinium cation is essentially planar, with a maximum diviation of 0.007 (6) Å for atom C3. The bond lengths (Allen *et al.*, 1987) and angles are normal.

In the crystal packing (Fig. 2), the protonated N1 atom and a nitrogen atom of the 2-amino group (N2) are hydrogen-bonded to the carboxylate oxygen atoms (O1 and O2) *via* a pair of intermolecular N1—H1N1···O2^i^ and N2—H1N2···O1^i^ hydrogen bonds (symmetry codes in Table 1), forming a ring motif of *R*_2_^2^(8) (Bernstein *et al.*, 1995). There is also an intramolecular O3—H1O3···O2 hydrogen bond in the 5-chlorosalicylate anion, which generates an S(6) ring motif. This motif is also observed in the crystal structure of 5-chloro-2-hydroxybenzoic acid (Raza *et al.*, 2010). The crystal structure is further stabilized by N2—H2N2···O1^ii^ and C8—H8A···O3^iii^ intermolecular interactions. These interactions have resulted in a molecular layer parallel to the (101) plane.

### Experimental

Hot methanol solutions (20 ml) of 2-amino5-methylpyridine (54 mg, Aldrich) and 5-chlorosalicylic acid (43 mg, Aldrich) were mixed and warmed over a heating magnetic stirrer hotplate for a few minutes. The resulting solution was allowed to cool slowly at room temperature and crystals of the title compound (I) appeared after a few days.

### Refinement

O- and N-bound H atoms were located in a difference Fourier maps. Atoms H1O3, H1N1 and H2N2 were refined freely, while atom H1N2 was refined with a bond length restraint N—H = 0.85 (1) Å [refined distance: O3—H1O3 = 0.92 (7) Å, N1—H1N1 = 0.94 (6) Å, N2—H1N2 = 0.853 (10) Å and N2—H2N2 = 0.85 (7) Å]. The remaining hydrogen atoms were positioned geometrically (C—H = 0.95–0.98 Å) and were refined using a riding model, with *U*_iso_(H) = 1.2*U*_eq_(C) or 1.5*U*_eq_(methyl C). A rotating group model was used for the methyl group.

### Crystal data

**Table:**

C_6_H_9_N_2_^+^·C_7_H_4_ClO_3_^−^	*F*(000) = 292
*M**_r_* = 280.70	*D*_x_ = 1.423 Mg m^−^^3^
Monoclinic, *P*2_1_	Mo *K*α radiation, λ = 0.71073 Å
Hall symbol: P 2yb	Cell parameters from 1722 reflections
*a* = 9.004 (7) Å	θ = 2.8–29.7°
*b* = 5.767 (5) Å	µ = 0.30 mm^−^^1^
*c* = 12.617 (10) Å	*T* = 100 K
β = 90.415 (16)°	Block, colourless
*V* = 655.2 (9) Å^3^	0.46 × 0.18 × 0.07 mm
*Z* = 2	

### Data collection

**Table:**

Bruker SMART APEXII Duo CCD area-detector diffractometer	2204 independent reflections
Radiation source: fine-focus sealed tube	1539 reflections with *I* > 2σ(*I*)
Graphite monochromator	*R*_int_ = 0.061
φ and ω scans	θ_max_ = 25.0°, θ_min_ = 1.6°
Absorption correction: multi-scan (*SADABS*; Bruker, 2009)	*h* = −10→10
*T*_min_ = 0.877, *T*_max_ = 0.979	*k* = −6→6
4785 measured reflections	*l* = −14→15

### Refinement

**Table:**

Refinement on *F*^2^	Secondary atom site location: difference Fourier map
Least-squares matrix: full	Hydrogen site location: inferred from neighbouring sites
*R*[*F*^2^ > 2σ(*F*^2^)] = 0.073	H atoms treated by a mixture of independent and constrained refinement
*wR*(*F*^2^) = 0.201	*w* = 1/[σ^2^(*F*_o_^2^) + (0.1249*P*)^2^] where *P* = (*F*_o_^2^ + 2*F*_c_^2^)/3
*S* = 1.01	(Δ/σ)_max_ = 0.001
2204 reflections	Δρ_max_ = 0.47 e Å^−^^3^
189 parameters	Δρ_min_ = −0.44 e Å^−^^3^
2 restraints	Absolute structure: Flack (1983), 941 Friedel pairs
Primary atom site location: structure-invariant direct methods	Flack parameter: 0.09 (16)

### Special details

**Table:**

Experimental. The crystal was placed in the cold stream of an Oxford Cryosystems Cobra open-flow nitrogen cryostat (Cosier & Glazer, 1986) operating at 100.0 (1) K.
Geometry. All e.s.d.'s (except the e.s.d. in the dihedral angle between two l.s. planes) are estimated using the full covariance matrix. The cell e.s.d.'s are taken into account individually in the estimation of e.s.d.'s in distances, angles and torsion angles; correlations between e.s.d.'s in cell parameters are only used when they are defined by crystal symmetry. An approximate (isotropic) treatment of cell e.s.d.'s is used for estimating e.s.d.'s involving l.s. planes.
Refinement. Refinement of *F*^2^ against ALL reflections. The weighted *R*-factor *wR* and goodness of fit *S* are based on *F*^2^, conventional *R*-factors *R* are based on *F*, with *F* set to zero for negative *F*^2^. The threshold expression of *F*^2^ > σ(*F*^2^) is used only for calculating *R*-factors(gt) *etc*. and is not relevant to the choice of reflections for refinement. *R*-factors based on *F*^2^ are statistically about twice as large as those based on *F*, and *R*- factors based on ALL data will be even larger.

### Fractional atomic coordinates and isotropic or equivalent isotropic displacement parameters (Å^2^)

**Table:**

	*x*	*y*	*z*	*U*_iso_*/*U*_eq_	
Cl1	0.49320 (17)	0.7884 (3)	0.59756 (12)	0.0882 (6)	
O1	0.1352 (4)	0.0724 (8)	0.6317 (2)	0.0718 (11)	
O2	0.1487 (4)	−0.0555 (7)	0.7972 (2)	0.0665 (10)	
O3	0.3088 (5)	0.1483 (9)	0.9319 (3)	0.0809 (13)	
N1	0.9568 (5)	0.6055 (9)	0.7468 (3)	0.0581 (11)	
N2	0.9360 (7)	0.7221 (10)	0.5745 (4)	0.0730 (15)	
C1	0.8976 (5)	0.5709 (10)	0.6496 (3)	0.0555 (13)	
C2	0.8031 (5)	0.3909 (10)	0.6344 (4)	0.0613 (15)	
H2A	0.7593	0.3646	0.5667	0.074*	
C3	0.7716 (6)	0.2485 (12)	0.7170 (4)	0.0724 (17)	
H3A	0.7049	0.1232	0.7056	0.087*	
C4	0.8336 (6)	0.2786 (13)	0.8183 (4)	0.0683 (14)	
C5	0.9247 (6)	0.4608 (11)	0.8286 (4)	0.0622 (14)	
H5A	0.9688	0.4902	0.8959	0.075*	
C6	0.8003 (8)	0.1215 (15)	0.9103 (5)	0.093 (2)	
H6A	0.8451	0.1854	0.9750	0.139*	
H6B	0.8416	−0.0328	0.8966	0.139*	
H6C	0.6925	0.1096	0.9191	0.139*	
C7	0.3500 (5)	0.2962 (12)	0.8536 (3)	0.0604 (13)	
C8	0.4479 (6)	0.4684 (11)	0.8751 (4)	0.0681 (16)	
H8A	0.4865	0.4834	0.9450	0.082*	
C9	0.4933 (6)	0.6238 (11)	0.7977 (4)	0.0666 (15)	
H9A	0.5615	0.7446	0.8138	0.080*	
C10	0.4360 (5)	0.5971 (11)	0.6960 (4)	0.0620 (13)	
C11	0.3378 (6)	0.4262 (11)	0.6724 (4)	0.0581 (13)	
H11A	0.3005	0.4116	0.6021	0.070*	
C12	0.2916 (5)	0.2732 (11)	0.7495 (3)	0.0507 (11)	
C13	0.1848 (5)	0.0861 (11)	0.7244 (3)	0.0579 (13)	
H1N1	1.019 (6)	0.735 (11)	0.754 (4)	0.070 (17)*	
H1N2	0.985 (6)	0.846 (7)	0.587 (5)	0.09 (3)*	
H2N2	0.888 (7)	0.705 (14)	0.517 (5)	0.09 (2)*	
H1O3	0.225 (7)	0.071 (13)	0.909 (5)	0.083 (19)*	

### Atomic displacement parameters (Å^2^)

**Table:**

	*U*^11^	*U*^22^	*U*^33^	*U*^12^	*U*^13^	*U*^23^
Cl1	0.0888 (9)	0.0908 (11)	0.0850 (10)	−0.0173 (9)	0.0112 (7)	0.0067 (10)
O1	0.076 (2)	0.099 (3)	0.0404 (16)	−0.016 (2)	−0.0140 (14)	−0.001 (2)
O2	0.076 (2)	0.080 (3)	0.0430 (16)	−0.011 (2)	−0.0066 (14)	0.0066 (19)
O3	0.103 (3)	0.103 (4)	0.0371 (17)	−0.013 (3)	−0.0145 (17)	0.0037 (19)
N1	0.066 (2)	0.068 (3)	0.0402 (19)	−0.005 (2)	−0.0016 (16)	−0.002 (2)
N2	0.095 (3)	0.082 (4)	0.042 (2)	−0.012 (3)	−0.009 (2)	−0.009 (2)
C1	0.058 (3)	0.070 (4)	0.038 (2)	0.004 (3)	0.0016 (19)	−0.005 (3)
C2	0.067 (3)	0.073 (4)	0.044 (2)	−0.004 (3)	−0.003 (2)	−0.010 (3)
C3	0.070 (3)	0.077 (5)	0.070 (3)	−0.010 (3)	0.004 (2)	−0.014 (3)
C4	0.067 (3)	0.079 (4)	0.059 (3)	0.004 (3)	0.007 (2)	−0.002 (3)
C5	0.076 (3)	0.072 (4)	0.038 (2)	0.003 (3)	0.001 (2)	0.002 (2)
C6	0.108 (5)	0.098 (5)	0.072 (3)	−0.003 (4)	0.014 (3)	0.017 (4)
C7	0.058 (3)	0.083 (4)	0.040 (2)	0.003 (3)	−0.0017 (18)	−0.006 (3)
C8	0.070 (3)	0.087 (5)	0.047 (2)	−0.002 (3)	−0.007 (2)	−0.016 (3)
C9	0.063 (3)	0.068 (4)	0.069 (3)	0.000 (3)	0.003 (2)	−0.018 (3)
C10	0.053 (2)	0.075 (4)	0.058 (3)	−0.005 (3)	0.005 (2)	−0.004 (3)
C11	0.064 (3)	0.069 (3)	0.042 (2)	0.003 (3)	0.0054 (19)	−0.008 (2)
C12	0.048 (2)	0.068 (3)	0.036 (2)	0.010 (3)	−0.0010 (15)	−0.009 (3)
C13	0.060 (3)	0.069 (4)	0.044 (2)	0.000 (3)	−0.0012 (19)	0.005 (3)

### Geometric parameters (Å, º)

**Table:**

Cl1—C10	1.742 (6)	C4—C5	1.339 (9)
O1—C13	1.251 (5)	C4—C6	1.504 (9)
O2—C13	1.273 (6)	C5—H5A	0.9500
O3—C7	1.359 (7)	C6—H6A	0.9800
O3—H1O3	0.92 (7)	C6—H6B	0.9800
N1—C1	1.349 (6)	C6—H6C	0.9800
N1—C5	1.359 (7)	C7—C8	1.354 (9)
N1—H1N1	0.94 (6)	C7—C12	1.417 (6)
N2—C1	1.335 (7)	C8—C9	1.389 (8)
N2—H1N2	0.853 (10)	C8—H8A	0.9500
N2—H2N2	0.85 (7)	C9—C10	1.388 (7)
C1—C2	1.355 (8)	C9—H9A	0.9500
C2—C3	1.358 (8)	C10—C11	1.356 (8)
C2—H2A	0.9500	C11—C12	1.380 (8)
C3—C4	1.403 (8)	C11—H11A	0.9500
C3—H3A	0.9500	C12—C13	1.478 (8)
			
C7—O3—H1O3	107 (4)	C4—C6—H6C	109.5
C1—N1—C5	120.9 (5)	H6A—C6—H6C	109.5
C1—N1—H1N1	116 (3)	H6B—C6—H6C	109.5
C5—N1—H1N1	123 (3)	C8—C7—O3	119.7 (4)
C1—N2—H1N2	124 (4)	C8—C7—C12	119.4 (5)
C1—N2—H2N2	114 (5)	O3—C7—C12	120.9 (5)
H1N2—N2—H2N2	121 (7)	C7—C8—C9	121.8 (4)
N2—C1—N1	116.5 (5)	C7—C8—H8A	119.1
N2—C1—C2	124.5 (4)	C9—C8—H8A	119.1
N1—C1—C2	119.0 (5)	C10—C9—C8	118.0 (5)
C1—C2—C3	119.3 (5)	C10—C9—H9A	121.0
C1—C2—H2A	120.4	C8—C9—H9A	121.0
C3—C2—H2A	120.4	C11—C10—C9	121.4 (5)
C2—C3—C4	122.7 (6)	C11—C10—Cl1	120.0 (4)
C2—C3—H3A	118.6	C9—C10—Cl1	118.6 (5)
C4—C3—H3A	118.6	C10—C11—C12	120.6 (4)
C5—C4—C3	115.1 (5)	C10—C11—H11A	119.7
C5—C4—C6	121.6 (5)	C12—C11—H11A	119.7
C3—C4—C6	123.3 (6)	C11—C12—C7	118.8 (5)
C4—C5—N1	122.9 (5)	C11—C12—C13	121.0 (4)
C4—C5—H5A	118.6	C7—C12—C13	120.2 (5)
N1—C5—H5A	118.6	O1—C13—O2	122.9 (5)
C4—C6—H6A	109.5	O1—C13—C12	118.3 (4)
C4—C6—H6B	109.5	O2—C13—C12	118.8 (4)
H6A—C6—H6B	109.5		
			
C5—N1—C1—N2	−179.9 (5)	C8—C9—C10—Cl1	−179.3 (4)
C5—N1—C1—C2	1.0 (7)	C9—C10—C11—C12	0.2 (8)
N2—C1—C2—C3	−179.7 (5)	Cl1—C10—C11—C12	179.9 (4)
N1—C1—C2—C3	−0.7 (8)	C10—C11—C12—C7	−0.8 (7)
C1—C2—C3—C4	−0.3 (9)	C10—C11—C12—C13	−179.9 (5)
C2—C3—C4—C5	1.0 (9)	C8—C7—C12—C11	0.8 (7)
C2—C3—C4—C6	−179.7 (6)	O3—C7—C12—C11	−179.4 (5)
C3—C4—C5—N1	−0.7 (8)	C8—C7—C12—C13	179.9 (5)
C6—C4—C5—N1	180.0 (5)	O3—C7—C12—C13	−0.3 (7)
C1—N1—C5—C4	−0.3 (8)	C11—C12—C13—O1	−1.3 (7)
O3—C7—C8—C9	180.0 (5)	C7—C12—C13—O1	179.6 (5)
C12—C7—C8—C9	−0.2 (8)	C11—C12—C13—O2	177.8 (4)
C7—C8—C9—C10	−0.4 (8)	C7—C12—C13—O2	−1.2 (7)
C8—C9—C10—C11	0.4 (8)		

### Hydrogen-bond geometry (Å, º)

**Table:**

*D*—H···*A*	*D*—H	H···*A*	*D*···*A*	*D*—H···*A*
O3—H1*O*3···O2	0.92 (6)	1.73 (7)	2.512 (6)	141 (6)
N1—H1*N*1···O2^i^	0.94 (6)	1.76 (6)	2.683 (7)	166 (5)
N2—H1*N*2···O1^i^	0.85 (5)	1.96 (5)	2.793 (8)	165 (4)
N2—H2*N*2···O1^ii^	0.85 (6)	2.04 (7)	2.811 (6)	152 (7)
C8—H8*A*···O3^iii^	0.95	2.58	3.425 (7)	148

Symmetry codes: (i) *x*+1, *y*+1, *z*; (ii) −*x*+1, *y*+1/2, −*z*+1; (iii) −*x*+1, *y*+1/2, −*z*+2.
